# An Improved Total Uncertainty Measure in the Evidence Theory and Its Application in Decision Making

**DOI:** 10.3390/e22040487

**Published:** 2020-04-24

**Authors:** Miao Qin, Yongchuan Tang, Junhao Wen

**Affiliations:** School of Big Data and Software Engineering, Chongqing University, Chongqing 401331, China; 20171979@cqu.edu.cn (M.Q.); jhwen@cqu.edu.cn (J.W.)

**Keywords:** Dempster–Shafer evidence theory, uncertainty measure, belief entropy, conflict management, decision making

## Abstract

Dempster–Shafer evidence theory (DS theory) has some superiorities in uncertain information processing for a large variety of applications. However, the problem of how to quantify the uncertainty of basic probability assignment (BPA) in DS theory framework remain unresolved. The goal of this paper is to define a new belief entropy for measuring uncertainty of BPA with desirable properties. The new entropy can be helpful for uncertainty management in practical applications such as decision making. The proposed uncertainty measure has two components. The first component is an improved version of Dubois–Prade entropy, which aims to capture the non-specificity portion of uncertainty with a consideration of the element number in frame of discernment (FOD). The second component is adopted from Nguyen entropy, which captures conflict in BPA. We prove that the proposed entropy satisfies some desired properties proposed in the literature. In addition, the proposed entropy can be reduced to Shannon entropy if the BPA is a probability distribution. Numerical examples are presented to show the efficiency and superiority of the proposed measure as well as an application in decision making.

## 1. Introduction

Uncertain information processing is a hot topic in intelligent information processing of theory and applications [1,2,3]. Many theories have been proposed to address the problem such as fuzzy set theory [4], probability theory [5], Dempster–Shafer evidence theory (DS theory) [6,7], etc. First proposed by Arthur Dempster and Glenn Shafer, Dempster–Shafer theory has been widely used in many fields such as risk evaluation [8,9,10], sensor data fusion [11], decision making [12,13], image recognition [14], classification [15,16], clustering [17,18] and so on [19,20]. However, some open issues remain unresolved. First, the fused information based on Dempster combination rule may generate counterintuitive results if evidence is highly conflicted with each other [21,22,23]. Second, many properties have been built to evaluate different uncertainty measures [24,25], but the rationality for some of these properties is still questionable. Third, during the process of calculating entropy, some characteristics of the basic probability assignment are lost. Fourth, the evidence in the open world assumption needs further consideration [26,27]. The uncertainty measure including belief entropy can be a promising method for uncertain information modeling, processing, and management of conflict in DS theory [28,29].

Uncertainty measure in DS theory has attracted much attention [30,31,32,33]. Over the years, many theories have been developed to quantify the uncertainty in DS Theory, such as Deng entropy [28], Pouly et al. method [34], Yager’s interval valued entropy [35], the cross entropy in Dempster–Shafer theory [36], uncertainty measure for *D* numbers [37], and so on [38,39]. To develop a reasonable measure, several properties for entropy of BPAs in the DS theory were built by Jirousek and Shenoy as an improved version of five axiomatic requirements in Shannon entropy [25,40]. The desired properties are: consistency with DS theory semantics, non-negativity, maximum entropy, monotonicity, probability consistency, additivity, and subadditivity. However, none of the existing measures satisfies all seven properties. The properties of belief entropy are still an open issue.

In this paper, we first analyze the rationality of some properties suggested in [24,40] and list seven reasonable properties, and then propose an improved total uncertainty measure in the evidence theory to measure the uncertainty of BPA. The hypotheses are that: (1) BPA is a generalization of probability; and (2) Shannon entropy can be adopted to DS theory. Thus, the new belief entropy can be a helpful tool for measuring uncertain degree of information. The proposed belief entropy has two components. The first component is an improved version of Dubois–Prade entropy [24], which can capture the non-specificity portion of uncertainty with a consideration of the size of frame of discernment (FOD). The second component is adopted from Nguyen entropy [41], which captures conflict in BPA. We show that our entropy satisfies six of seven desired properties. In addition, our entropy can reduce to Shannon entropy if a BPA is degenerated to a probability distribution. The proposed method is applied in decision making, which follows the works in diverse applications [42,43].

The rest of this paper is outlined as follows. In Section 2, the preliminaries of DS theory and entropy are briefly introduced. The improved entropy is presented in Section 3, as well as the analysis of the properties. In Section 4, some numerical examples are carried out to demonstrate the effectiveness and superiority of the proposed method. In Section 5, the proposed entropy is used in decision making. Finally, the conclusion and a discussion of future work are given in Section 6.

## 2. Preliminaries

Some basic preliminaries are introduced in this section.

### 2.1. Dempster–Shafer Evidence Theory

Dempster–Shafer evidence theory is widely applied in addressing uncertainties [44,45]. Some basic concepts are introduced as follows [6,7].

Let *X* be a set of mutually exclusive and exhaustive events, denoted as:

(1) $$X=\{\theta_{1},\theta_{2},\theta_{3}\ldots \theta_{\left|X\right|}\},$$

where the set *X* is named the frame of discernment (FOD). The power set of *X* is defined as follows:

(2) $$2^{X}=\{\emptyset ,\left\{\theta_{1}\right\}\ldots \left\{\theta_{\left|X\right|}\right\},\left\{\theta_{1},\theta_{2}\right\}\ldots \left\{\theta_{1},\theta_{2}\ldots \theta_{i}\right\}\ldots |X|\},$$

For a FOD $X=\{\theta_{1},\theta_{2},\theta_{3}\ldots \theta_{\left|X\right|}\}$, the mass function is a mapping *m* from $2^{X}$ to [0,1], defined as follows:

(3) $$m:2^{X}\to \left[0,1\right],$$

which satisfies the following condition:

(4) $$m\left\{\emptyset \right\}=0\;and\;\sum_{A\in 2^{X}}m\left(A\right)=1.$$

In DS theory, the mass function is also referred as basic probability assignment (BPA). If a subset *a* satisfies $a\in 2^{X}$ and m(a)>0, then *a* is called the focal element of *m*. If a BPA *m* contains a focal element *X* with belief 1, then the BPA *m* is a vacuous BPA.

### 2.2. Dempster’s Rule of Combination

Assume there are two independent BPAs $m_{1}$ and $m_{2}$; the Dempster’s rule of combination, which is denoted as $m=m_{1}⨁m_{2}$, is defined as: follows [6]:

(5) $$m\left(A\right)=\left\{\begin{array}{cc} \frac{1}{1-K}\sum_{B⋂C=A}m_{1}\left(B\right)m_{2}\left(C\right), & A\neq \emptyset ,\\ 0, & A=\emptyset , \end{array}\right.$$

where the *K* is is a normalization constant defined as follows:

(6) $$K=\sum_{B⋂C=\emptyset }m_{1}\left(B\right)m_{2}\left(C\right)$$

The normalization constant *K* is assumed to be non-zero. if K=0, then $m_{1}$ and $m_{2}$ are in total-conflict and cannot be combined using Dempster’s rule. If k=1, $m_{1}$ and $m_{2}$ are non-interactive with each other, then $m_{1}$ and $m_{2}$ are non-conflicting.

The Dempster’s rule of combination combines two BPAs in such a way that the new BPA represents a consensus of the contributing pieces of evidence. It also focus BPA on single set to decrease the uncertainty in the system based on the combination rule, which can be useful in decision making process.

### 2.3. Belief Entropy in Dempster–Shafer Evidence Theory

Several methods have been proposed to solve the problem of uncertainty measure in DS theory. Some previous methods are briefly summarized in Table 1.

## 3. The Improved Total Uncertainty Measure

### 3.1. The New Measure

To address the uncertainty in the FOD, an improved total uncertainty measure is proposed in this paper. The improved total uncertainty measure denoted as $Q_{(}m)$ is defined as follows:

(7) $$Q_{(}m)=\sum_{A\subseteq X}\frac{\left|A\right|}{\left|X\right|}m\left(A\right)log_{2}\left(\left|A\right|\right)+\sum_{A\subseteq X}m\left(A\right)log_{2}\left(\frac{1}{m(A)}\right),$$

where $\left|A\right|$ denotes the cardinality of the focal element *A*, $\left|X\right|$ is the number of element within *X*, and *m* is the BPA in FOD *X*. The first component $\sum_{A\subseteq X}\frac{\left|A\right|}{\left|X\right|}m\left(A\right)log\left(\left|A\right|\right)$ is designed as a weighted measure for the total non-specificity among focal elements. The second component $\sum_{A\subseteq X}m\left(A\right)log_{2}\left(\frac{1}{m(A)}\right)$ can be interpreted as a portion to capture the uncertainty in the form of conflict. The improved total uncertainty measure also guarantees that the measure could be reduced to Shannon entropy in Bayesian probability cases. Notice that the coefficient $\frac{\left|A\right|}{\left|X\right|}$ is added to consider the size of FOD. As shown in Table 1, the number of elements contained in FOD is not included for most measures.

### 3.2. Desired Properties of Belief Entropy

In this paper, we consider some properties of entropy H(m) (*m* is a BPA) proposed in [40,51].

Let *X* and *Y* denote random variables with state spaces $\Omega_{X}$ and $\Omega_{Y}$, respectively. Let $m_{X}$ and $m_{Y}$ denote distinct BPAs for X and Y, respectively. Let $\gamma_{X}$ and $\gamma_{Y}$ denote the vacuous BPAs for *X* and *Y*, respectively.

(1) Consistency with DS Theory semantics. If a definition of entropy of *m*, or a portion of a definition, is based on a transform of BPA *m* to a probability mass function (PMF) $p_{m}$, then the transform must satisfy the following condition: $P_{m_{1}⨁m_{2}}=P_{m_{1}}⊗P_{m_{2}}$

(2) Non-negativity. $H_{m_{X}}⩾0$, with equality if and only if there is a $x\in \Omega_{X}$ where *x* is a single element set that satisfies $m_{X}\left(\left\{x\right\}\right)=1$. It is similar to probabilistic case.

(3) Monotonicity. If $\left|\Omega_{X}\right|<\left|\Omega_{Y}\right|$, then $H\left(\gamma_{X}\right)<H\left(\gamma_{Y}\right)$.

(4) Probability consistency. If $m_{X}$ is a Bayesian BPA for *X*, then $H\left(m_{X}\right)=\sum_{x\subseteq \Omega_{X}}m_{X}\left(\left\{x\right\}\right)log\frac{1}{m_{X}\left(\left\{x\right\}\right)}$. In other words, in the case of Bayesian BPA, the entropy reduces to Shannon entropy [52].

(5) Maximum entropy. $H\left\{m_{X}\right\}⩽H\left\{\gamma_{X}\right\}$, with equality if and only if $m_{X}=\gamma_{X}$. The entropy in [40] shows that it is rational that the vacuous BPA $\gamma_{X}$ has the most uncertainty among all cases since in this case $\gamma_{X}\left(\Omega_{X}\right)=1$. One hundred percent belief degree is assigned to $\gamma_{X}\left(\Omega_{X}\right)$, which provides no information to help with decision making because the belief of each proposition in FOD is completely unknown.

(6) Additivity. Given two distinct BPAs $m_{X}$ and $m_{Y}$ for *X* and *Y*, we can combine them using Dempster’s rule, denoted as $m_{X}⊕m_{Y}$. Then, $H(m_{X}⊕m_{Y})$ must satisfy the following equation:

(8) $$H\left(m_{X}⊕m_{Y}\right)=H\left(m_{X}\right)+H\left(m_{Y}\right).$$

(7) Set consistency. $H\left(m\right)=log_{2}\left(|a|\right)$ whenever *m* is deterministic with focal set a, i.e., m(a)=1.

(8) Range. For any BPA $m_{X}$ for *X*, $0\leq H\left(m\right)\leq log_{2}\left|\Omega_{X}\right|$.

(9) Subadditivity. Suppose *m* is a BPA for $\left\{X,Y\right\}$; then, with marginal BPA $m_{X}^{\prime }$ for *X* and $m_{Y}^{\prime }$ for *Y*, we have

(9) $$H\left(m\right)\leq H\left(m_{X}^{\prime }\right)+H\left(m_{Y}^{\prime }\right)$$

It is argued in [40] that, if a definition of entropy of m, or a portion of a definition, is based on a transform of BPA to a PMF, then the transform must satisfy the condition $P_{m_{1}⊕m_{2}}=P_{m_{1}}⊗P_{m_{2}}$ where ⊗ is the combination rule in probability theory, and ⊕, as mentioned in Section 2, is Dempster’s combination rule. Notice that only *if* a transform is used, then it must be consistent with Dempster’s rule. Since none of the methods except for Jirousek–Shenoy Equation in Table 1 use transform of BPA to a PMF, we do not discuss the Consistency with DS Theory semantics property in this article.

The Set consistency property requires that $H\left(\gamma_{X}\right)=log_{2}\left|\Omega_{X}\right|$. The probability consistency property would require that, for the Bayesian uniform BPA $m_{u}$, $H\left(m_{u}\right)=log_{2}\left|\Omega_{X}\right|$ as well. Thus, these two requirements would entail that $H\left(\gamma_{X}\right)=H\left(m_{u}\right)$. On the contrary, the Maximum entropy property indicates that the entropy of vacuous BPA $H(\gamma_{X})$ should be maximum, $H\left(\gamma_{X}\right)>H\left(m_{u}\right)$. Before further analysis of these two properties, first consider the following the example.

**Example** **1.** *Suppose there is a race with three bikes. We have two experts that make following the statements.*

- *Expert 1: “The three bikes and riders are similar”.*
- *Expert 2: “I do not have information about the characteristics of each bike and rider”.*

If we represent these information in BPA, the opinion of Expert 1 produces a uniform distribution ($\frac{1}{3}$, $\frac{1}{3}$, $\frac{1}{3}$) and the second one a vacuous BPA. The question is, if we must bet on a bike that will win this race, on which should we place our bet following the information of these experts? In this case, we do not have anything that allows us to bet on a bike, thus our final decision will be made randomly. Hence, the information, or uncertainty, should be the same. In both cases, it must reach the maximum uncertainty value. Therefore in this paper, we modify the Maximum entropy property instead of adopting the original property:

- Maximum entropy. $H\left(m_{X}\right)⩽H\left(\gamma_{X}\right)$, with equality if and only if $m_{X}=\gamma_{X}$ or $m_{X}$ is a uniform distribution.

The Range property requires that, for all BPAs, the value of entropy should be bounded by $log_{2}\left|\Omega_{X}\right|$; we disagree. In Shannon’s information theory, the maximum number of bits required to represent the uncertainty of a system with *n* status is $log_{2}\left(n\right)$, which is reasonable since the system can be represented only by state number *n*. However, in this case, the BPAs focus on several subsets (up to $2^{n}-1$) and each of them can have non-empty intersection with others. However, because of the non-specificity part of total uncertainty, one cannot even say that the total uncertainty should be bounded by $log_{2}\left(2^{n}-1\right)$. Based on this analysis, we do not adopt Range property.

Based on the analysis above, we list seven properties that may be satisfied by uncertainty measure in DS theory:

- Non-negativity
- Monotonicity
- Probability consistency
- Maximum entropy
- Additivity
- Set consistency
- Subadditivity

### 3.3. Property of the New Measure

The analysis of the property for the proposed measure is presented as follows.

(1) ***Non-negativity***. The first component $\sum_{A\subseteq X}\frac{\left|A\right|}{\left|X\right|}m\left(A\right)log_{2}\left(\left|A\right|\right)=0$ and second component $\sum_{A\subseteq X}m\left(a\right)log_{2}\left(\frac{1}{m(A)}\right)=0$ if and only if there exists x⊆X and $m(\left\{x\right\})=1$. For all BPAs, $\frac{\left|A\right|}{\left|X\right|}>0$, then Q(m)>0. Therefore, $Q_{(}m)$ satisfies the non-negativity property.

(2) ***Monotonicity***. Suppose a vacuous BPA $\gamma_{X}$; then, $Q\left(\gamma_{X}\right)=\left|X\right|$. Since it is monotonic in $\left|X\right|$, Q(m) satisfies the monotonicity property.

(3) ***Probability Consistency***. If *m* is Bayesian, then the first component $\sum_{A\subseteq X}\frac{\left|A\right|}{\left|X\right|}m\left(A\right)log_{2}\left(\left|A\right|\right)=0$. In this case, Q(m) is degenerated to Shannon entropy. Thus, Q(m) satisfies the probability consistency property.

(4) ***Maximum entropy***. Suppose a Bayesian uniform BPA $m_{u}$ and *n* denotes $\left|X\right|$; therefore, for each focal element, $m\left(A\right)=\frac{1}{n}$. Suppose another vacuous BPA $\gamma_{X}$ with *n* denoting $\left|X\right|$. After calculation, it is clear that the entropies of both cases reach the maximum value of $log_{2}n$ at the same time, thus Q(m) satisfies the Maximum entropy property.

(5) ***Additivity***. Let BPA $X=\left\{x_{1},x_{2},x_{3}\right\}$, $Y=\left\{y_{1},y_{2}\right\}$ and X×Y be the product space of *X* and *Y*. BPAs for *X* and *Y* are listed as follows:

$$\begin{array}{c} m_{X}\left\{x1,x2\right\}=0.6\\ m_{X}\left\{x3\right\}=0.3\\ m_{X}\left\{X\right\}=0.3\\ m_{Y}\left\{Y\right\}=1 \end{array}$$

Now, we build the BPA $m^{\prime }=m_{X}\times m_{Y}$ on X×Y. The BPA $m^{\prime }$ has the following masses:

$$\begin{array}{c} m^{\prime }\left(\left\{z_{11},z_{12},z_{21},z_{22}\right\}\right)=0.6\\ m^{\prime }\left(\left\{z_{31},z_{32}\right\}\right)=0.1\\ m^{\prime }\left(X\times Y\right)=0.3 \end{array}$$

where $z_{ij}=\left(x_{i},y_{j}\right)$.

The values of uncertainty via Q(m) are:

(10) $$\begin{array}{c} Q(m^{\prime })=2.9043\\ Q\left(m_{X}\right)+Q\left(m_{Y}\right)=3.1710 \end{array}$$

Clearly, Q(m) does not meet the requirements of Additivity property. The second component $\sum_{A\subseteq X}m\left(a\right)log_{2}\left(\frac{1}{m(A)}\right)$ satisfies the additivity property that the log of a product is the sum of the logs. Let *R* be the product space of *X*, *Y*, R=A×B⊆X×Y; then, $H\left(m\right)=\sum_{R\subseteq X\times Y}^{}m\left(R\right)log_{2}\left(\frac{1}{m(R)}\right)=\sum_{A\subseteq X,B\subseteq Y}^{}m\left(A\times B\right)log_{2}\left(\frac{1}{m(A\times B)}\right)=\sum_{A\subseteq X,B\subseteq Y}^{}m\left(A\right)m\left(B\right)log_{2}\left(\frac{1}{m(A)m(B)}\right)=\sum_{A\subseteq X}^{}m_{X}\left(A\right)log_{2}\left(\frac{1}{m_{X}\left(A\right)}\right)+\sum_{B\subseteq Y}^{}m_{Y}\left(B\right)log_{2}\left(\frac{1}{m_{Y}\left(B\right)}\right)=H\left(m_{X}\right)+H\left(m_{Y}\right)$. The first part $\sum_{A\subseteq X}\frac{\left|A\right|}{\left|X\right|}m\left(A\right)log\left(\left|A\right|\right)$ does not meet the requirement of additivity for adding the coefficient $\frac{\left|A\right|}{\left|X\right|}$. Thus, Q(m) does not satisfy the Additivity property. Notice that, in probability cases, Q(m) degenerates to Shannon entropy and the Additivity property holds. In addition, while both Deng’s entropy and Q(m) fail to satisfy this property, their performances shown in Section 4 and Section 5 are still reasonable.

(6) ***Set consistency***. For vacuous BPA $\gamma_{X}$ of *X*, Q(m) is reduced to Nguyen entropy, and, according to Table 1, the definition of Nguyen entropy satisfies the Set consistency property, thus Q(m) satisfies the Set consistency property.

(7) ***Subadditivity***. Consider BPA *m* for $\left\{X,Y\right\}$ as follows: $m\left\{\left(x,y\right),(\bar{x},\bar{y}\right\}=m\left\{\left(\bar{x},y\right),(x,\bar{y}\right\}=\frac{1}{2}$, for this BPA, Q(m)=1.5000. $m_{X}^{\prime }$ and $m_{X}^{\prime }$ have vacuous BPA $\gamma_{X}$ and $\gamma_{Y}$, $Q\left(m_{X}^{\prime }\right)+Q\left(m_{Y}^{\prime }\right)=2$, and the inequality holds when *m* reduces to a possibility distribution. Therefore, Q(m) satisfies the Subadditivity property.

As shown in Table 1, both Pal et al.’s equation and Q(m) satisfies six of seven properties listed in Section 3.2; the limitation of Pal et al.’s is discussed later in Section 4.

## 4. Numerical Examples

In this section, some typical numerical examples are presented to show the effectiveness of the proposed measure.

**Example** **2** (Adopted from [28])**.** *Given a frame of discernment X with 15 elements which are denoted as Elements 1–15, the BPA is shown as follows:*

$m\left(\left\{3,4,5\right\}\right)=0.05,m\left(\left\{6\right\}\right)=0.05,m\left(\left\{A\right\}\right)=0.8,m\left(\left\{X\right\}\right)=0.1.$

**Figure 1:**
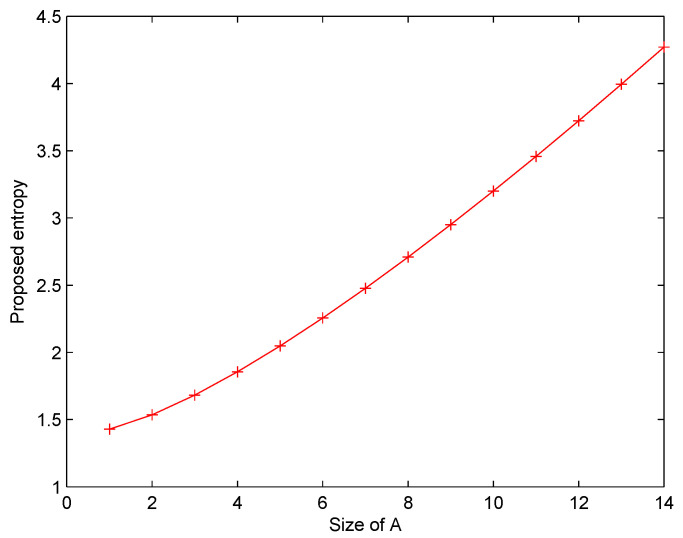
Q(m) changes with the variable *A*.

**Figure 2:**
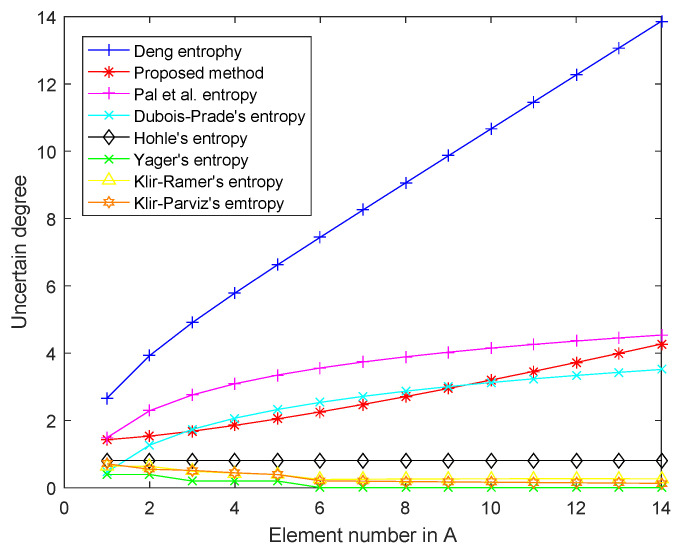
Uncertain degree with the proposed measure and other methods.

Table 2 and Figure 1 list the values of the proposed entropy Q(m) when *A* increases. Figure 2 shows that the uncertainty degree measured by the proposed measure increases along with the growing size of *A*.

It is rational since more information volume becomes unknown if the size of *A* rises.

Figure 2 lists the performance of other uncertainty measures including Dubois–Prade’s entropy [24], Höhle’s entropy [49], Yager’s entropy [48], Klir–Ramer’s entropy [46], Klir–Parviz’s entropy [49], and Pal et al.’s entropy [50]. According to Figure 2, only Deng entropy, Dubois–Prade’s entropy, Pal et al.’s entropy and the proposed entropy increase monotonously with the size of *A*. On the contrary, other measures either decrease irregularly or fluctuate as the size of *A* increases. This is because other methods do not consider the size of *A* and *X* simultaneously in the definition. Using more available information means less uncertainty.

**Example** **3** (Adopted from [53])**.** *Consider two different FODs* $\Theta_{1}=\left\{a,b,c,d\right\}$ *and* $\Theta_{2}=\left\{a,b,c\right\}$*; the BPAs are given as follows:*

$$\begin{array}{c} m_{1}:m_{1}\left(\left\{a,b\right\}\right)=0.4,\\ m_{1}\left(\left\{c,d\right\}\right)=0.6.\\ m_{2}:m_{2}\left(\left\{a,c\right\}\right)=0.4,\\ m_{2}\left(\left\{b,c\right\}\right)=0.6. \end{array}$$

According to the definitions in Table 1, the uncertainty of $m_{1}$ and $m_{2}$ can be calculated with different uncertainty measures; the results are shown in Table 3. The results calculated by Deng entropy, Pal et al.’s entropy, and Dubois–Prade entropy fail to show the difference of uncertain degree among the two bodies of evidence. The FOD of $m_{1}$ consists of four elements denoted as *a*, *b*, *c*, and *d*, while the FOD of $m_{2}$ only has three elements denoted as *a*, *b*, and *c*. It is expected that the uncertainty of these two BPAs should be different. Deng entropy, Pal et al.’s entropy, and Dubois–Prade entropy fail to measure the difference between these BPAs, while the proposed method can effectively measure the difference by considering the size of the FOD. The final result seems rational; although there fewer less elements in the second BPA $m_{2}$, the intersection in $m_{2}$ provides more uncertainty while all elements in $m_{1}$ are independent from each other. Therefore, the proposed method appears to be a reasonable way to measure uncertainty of evidence under such circumstances.

## 5. Application in Conflict Data Fusion

In this section, the proposed measure is applied to a case study on conflict data-based decision making. The dataset is adopted from [53,54].

### 5.1. Problem Statement

Supposing that the FOD is $\Theta =\left\{F_{1},F_{2},F_{3}\right\}$, which consists of three types of faults for the machines. The diagnosis sensors are denoted as $S=\left\{S_{1},S_{2},S_{3},S_{4},S_{5}\right\}$. Five sensors are positioned on different places for collecting diagnosis data. The results represented by BPAs are shown in Table 4.

### 5.2. Decision Making Procedure

**Figure 3:**
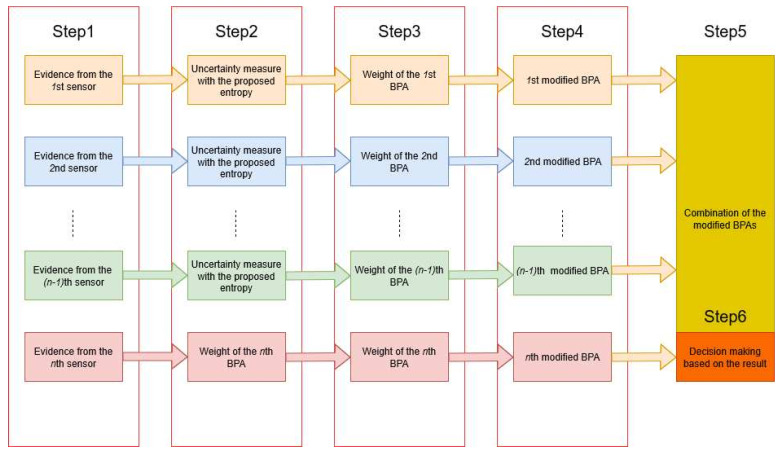
Decision making progress based on the improved total uncertainty measure.

The process for decision making based on the improved belief entropy is proposed in Figure 3. Six steps are designed as follows.

#### *Step* *1*

Data from sensors are modeled as BPAs. As shown in Table 4, each piece of evidence is modeled as a BPA.

#### *Step* *2*

Measure the uncertain degree using the improved total uncertainty measure in Equation (7). Generally, the more dispersive is the mass value assigned among the power set, the bigger is the entropy of the BPA. The entropy of each BPA is calculated as follows: $Q(m_{1})=1.5664$, $Q(m_{2})=0.4690$, $Q(m_{3})=1.4878$, $Q(m_{4})=1.5700$, and $Q(m_{5})=1.4955$. Notice that entropy $Q_{(}m_{2})$ is much smaller than the others because $m_{2}$ assigns a belief of 90% on $F_{2}$, while other BPAs are more dispersive.

#### *Step* *3*

Calculate the relative weight based on the uncertain degree of each evidence. It is commonly accepted that the bigger is the entropy, the higher is the uncertain degree. The relative weight of each BPA is defined according to the new belief entropy. For the *i*th BPA, the corresponding relative weight among all *n* BPAs is defined as follows:

(11) $$W_{BPA}\left(m_{i}\right)=\frac{Q(m_{i})}{\sum_{j=1}^{n}Q\left(m_{j}\right)}.$$

The relative weight of each BPA in Table 4 can be calculated with Equation (11). The calculation results are: $W_{BPA}\left(m_{1}\right)=0.2377$, $W_{BPA}\left(m_{2}\right)=0.0712$, $W_{BPA}\left(m_{3}\right)=0.2258$, $W_{BPA}\left(m_{4}\right)=0.2383$, and $W_{BPA}\left(m_{5}\right)=0.2270$.

#### *Step* *4*

Evidence modification for the original BPA using the proposed measure. By using the relative weight of each BPA, we unify the BPAs given by all sensors and generate one weighted BPA. The resulting weighted BPA is used in final data fusion. For a proposition *A*, the modified BPA can be derived according to the following function:

(12) $$m\left(A\right)=\sum_{i=1}^{n}m_{i}\left(A\right)W_{BPA}\left(m_{i}\right).$$

According to Equation (12), the BPAs in Table 4 are modified and the result is as follows: $m(\left\{F_{1}\right\})=0.4957$, $m(\left\{F_{2}\right\})=0.1953$, $m(\left\{F_{3}\right\})=0.0784$, $m(\left\{F_{1},F_{3}\right\})=0.2305$.

#### *Step* *5*

Evidence fusion using Dempster’s rule of combination in Equations (5) and (6) with (n−1) times based on the modified BPA. The final fusion result is as follows:

$$\;\begin{array}{c} \begin{array}{c} m\left(\left\{F_{1}\right\}\right)=\left(\left(\left(m⊕m\right)⊕m\right)⊕m\right)\left(\left\{F_{1}\right\}\right)=0.9849,\\ m\left(\left\{F_{2}\right\}\right)=\left(\left(\left(m⊕m\right)⊕m\right)⊕m\right)\left(\left\{F_{2}\right\}\right)=0.0014,\\ m\left(\left\{F_{3}\right\}\right)=\left(\left(\left(m⊕m\right)⊕m\right)⊕m\right)\left(\left\{F_{3}\right\}\right)=0.0105,\\ m\left(\left\{F_{1},F_{3}\right\}\right)=\left(\left(\left(m⊕m\right)⊕m\right)⊕m\right)\left(\left\{F_{1},F_{3}\right\}\right)=0.0032. \end{array} \end{array}$$

#### *Step* *6*

Decision-making based on data fusion results. From the original data in Table 4, the report from the second sensor is highly conflicted with the other sensors on $F_{1}$ and $F_{2}$. Based on all the data in Table 4, intuitively, $F_{1}$ should be recognized as the potential fault type. The fusion results with five sensors have a belief of over 98% on the potential fault type $\left\{F_{1}\right\}$.

### 5.3. Discussion

The result with different information fusion methods is shown in Table 5. The fusion results for fault type identification based on the proposed method with two, three, four, and five sensors are shown in Table 6.

**Figure 4:**
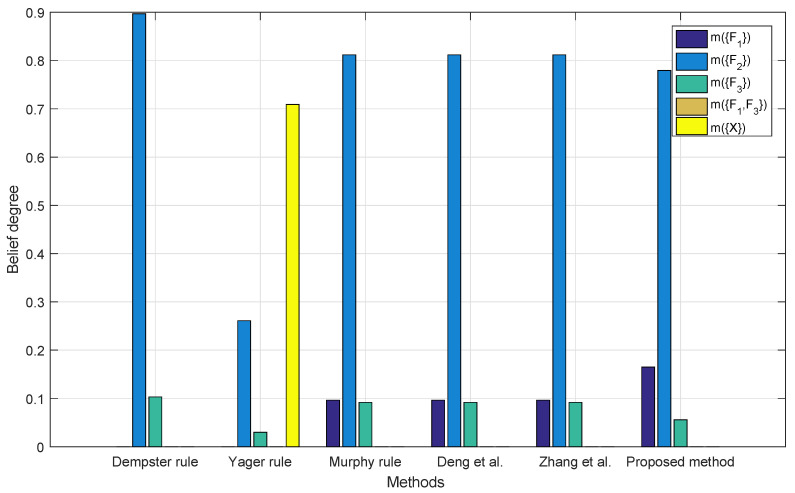
Fusion results with two BPAs.

Figure 4 shows the performance of different combination methods with two sensor reports. One cannot make a decision based on the Yager’s combination rule since, in this case, the universal set denoted as $m(\left\{X\right\})$ has the highest belief among all the propositions. Other methods have a belief of more than 80% on $F_{2}$, while according to the prior knowledge, the report of $m_{2}$ may come from a bad sensor and $F_{2}$ cannot be the potential fault. The result with the proposed method has the lowest belief on $F_{2}$. In this case, $m_{1}$ and $m_{2}$ are highly conflicted with each other, while $m_{1}\left(\left\{F_{1}\right\}\right)=0.41$, $m_{1}\left(\left\{F_{2}\right\}\right)=0.29$, $m_{1}\left(\left\{F_{3}\right\}\right)=0.30$, $m_{1}\left(\left\{F_{1},F_{3}\right\}\right)=0$ and $m_{2}\left(\left\{F_{1}\right\}\right)=0$; $m_{2}\left(\left\{F_{2}\right\}\right)=0.90$, $m_{2}\left(\left\{F_{1}\right\}\right)=0.10$, and $m_{2}\left(\left\{F_{1},F_{3}\right\}\right)=0$; and, clearly, $m_{2}\left(\left\{F_{2}\right\}\right)=0.90$ is much more influential than $m_{1}\left(\left\{F_{1}\right\}\right)=0.41$, which allows the decision making procedure to allocate too much weight on $m_{2}$ during Step 3, resulting in the failure of identifying the right target.

**Figure 5:**
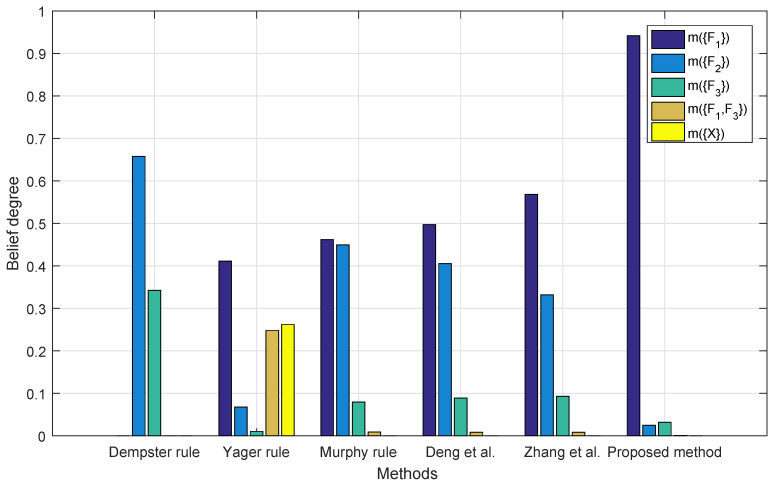
Fusion results with three BPAs.

Figure 5 shows the fusion results with three sensor reports. The result of Dempster’s rule seems counterintuitive for assigning the highest belief on $F_{2}$. The fusion result of other methods assign the highest belief on $F_{2}$; however, the proposed method is the only one that has over 90% on the right fault $F_{1}$ while none of other methods assign a belief of more than 60% on fault $F_{1}$. In this case, the proposed method successfully identify the right fault with only three sensor reports with the highest belief degree.

**Figure 6:**
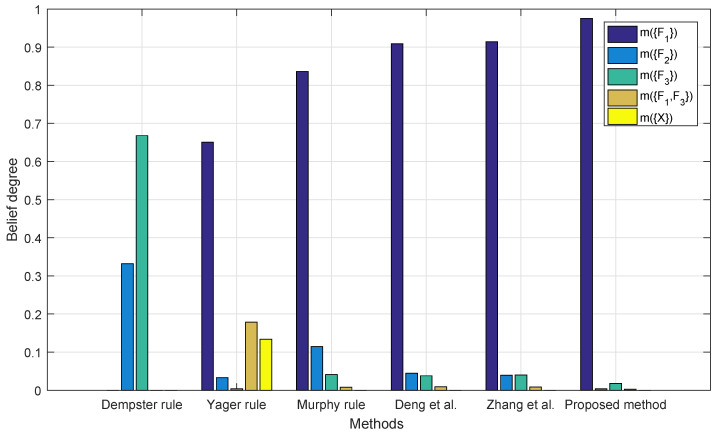
Fusion results with four BPAs.

As shown in Figure 6, the fusion result of Dempster’s combination rule with four BPAs still leads to the wrong fault type $F_{2}$ due to the conflicting report given by $m_{2}$, while the proposed method has the highest belief degree than the other methods on the right fault type $F_{1}$.

**Figure 7:**
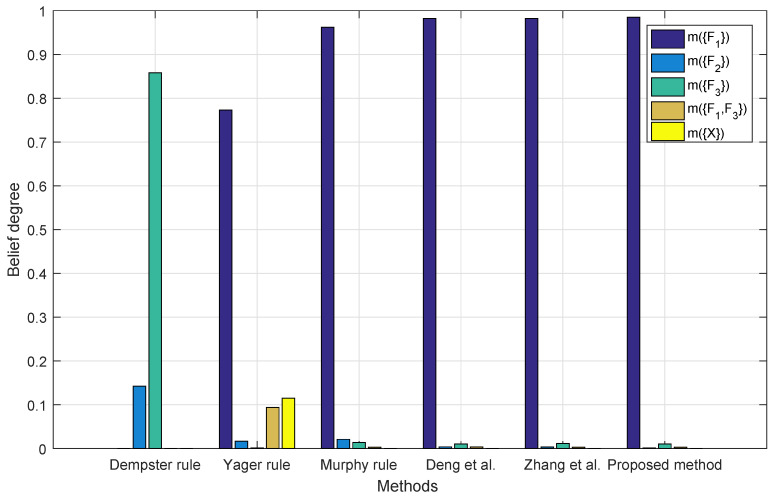
Fusion results with five BPAs.

The fusion results with all five sensors are shown in Figure 7; the proposed method has the highest belief of 98.49% on the right fault $F_{1}$.

Table 5 summarizes Figure 4, Figure 5, Figure 6 and Figure 7. For Dempster’s rule, the belief assigned to $F_{1}$ remains zero no matter how many BPAs are fused, but, intuitively, as shown in Table 4, $F_{1}$ should be identified as the target. For Yager’s rule, because of the belief assigned to $m(\left\{X\right\})$, the fusion result of five BPAs shows that only a belief of 77.32% is assigned to $F_{1}$, while other methods except for Dempster’s all have much better performance, with a belief of over 90% on $F_{1}$.

The fault type identification based on the proposed method with two, three, four, and five sensors is indicated in Table 6. As shown in the table and Figure 5, the proposed method is the only one that has over 90% on the right fault $F_{1}$ when only three sensor reports are given. Suppose that the data in Table 4 only consist of $m_{1}$, $m_{2}$, and $m_{3}$; based on our intuition and the provided information, $F_{1}$ should be the target, and the result shows that the proposed method indeed identifies $F_{1}$. Later fusion results with four and five BPAs also indicate that $F_{1}$ should be the right fault. From this point, the decision made based on the proposed method with limited number of reports has certain degree of validity.

According to the fusion results, the proposed method has several superiorities in comparison with the other methods. First, as shown in Section 4, the proposed method can effectively measure the uncertainty degree of two different BPAs even if the same belief value is assigned on different FODs, while both Deng entropy and the Dubois–Prade entropy are failed. Secondly, it is presented in Section 3 that Q(m) satisfies six of seven properties, ensuring that the fusion results in Table 5 are reasonable and consistent with our intuition. Even in special cases such as vacuous BPA or probability cases, Q(m) still presents rational result. In addition, as shown in Figure 5, the proposed method recognizes the right fault with limited number of reports while the other methods cannot make the right decision until there are four reports. The proposed method reduces the interference of conflicting evidence more efficiently than the other method due to the consideration on not only the uncertainty appearing in the mass function, but also the size of FOD and the size of each proposition. As shown in Table 1, only the proposed method considers the size of FOD. Finally, the proposed method is based on information volume measured by modified belief entropy, thus the physical meaning is clear.

There are several reasons that contribute to the performance of the decision-making procedure. First, all sensor data are preprocessed before the decision-making procedure. With the corresponding BPAs, our process successfully identifies the fault and eliminates the conflicting evidence by using the proposed method. The effectiveness and superiority proved in Section 3 and Section 4 also guarantee the efficiency of our decision-making approach. Finally, the relative weight is calculated using the proposed measure and the final result is based on the Dempster’s combination rule, which combines the merits of Dempster’s combination rule and the effectiveness of the proposed method.

## 6. Conclusions

An improved total uncertainty measure based on total non-specificity the uncertainty in the form of conflict is proposed in this paper. The rationality of some previous properties are discussed and seven desired properties are listed to define a meaningful measure. The new measure satisfies six of seven desired properties of belief entropy. The proposed entropy not only captures the non-specificity and conflict in uncertainty, but also considers the size of FOD and the size of the proposition with respect to the FOD. Numerical examples show that the proposed entropy can quantify the uncertain degree of the BPA more accurately than the other uncertainty measures when the same belief value is assigned to different FODs. In the case of vacuous BPA and uniform distribution, the proposed method improves the performance of other measures and provides a result that is consistent with our intuition.

A decision making approach based on the proposed measure was applied to a case study. The fusion result shows the superiorities and effectiveness of the improved method in comparison with the other methods. In future studies, the proposed method will be applied in more real word applications such as image compression, image recognition, etc.

## Figures and Tables

**Table 1:** Belief entropy in Dempster–Shafer evidence theory.

Name	Defination	Set Cons.	Non-Neg	Max. Ent.	Monton.	Prob. Cons.	Additivity	Subadditivity
Dubois–Prade Equation [24]	$H_{D}\left(m\right)=\sum_{A\subseteq X}m\left(a\right)log_{2}\left|A\right|$	yes	no	no	yes	no	yes	yes
Nguyen Equation [41]	$H_{n}\left(m\right)=\sum_{A\subseteq X}m\left(A\right)log_{2}\left(\frac{1}{m(A)}\right)$	no	no	no	no	yes	yes	no
Klir–Ramer Equation [46]	$H_{kr}\left(m\right)=-\sum_{A\subseteq X}m\left(A\right)log_{2}\sum_{B\subseteq X}m\left(B\right)\frac{\left|A\cap B\right|}{\left|B\right|}$	no	yes	no	yes	yes	yes	no
Klir–Parviz Equation [47]	$H_{kp}\left(m\right)=-\sum_{A\subseteq X}m\left(A\right)log_{2}\sum_{B\subseteq X}m\left(B\right)\frac{\left|A\cap B\right|}{\left|A\right|}$	no	yes	no	yes	yes	yes	no
Deng Equation [28]	$H_{D}\left(m\right)=\sum_{A\subseteq X}m\left(A\right)log_{2}\frac{2^{\left|A\right|}-1}{m(A)}$	no	yes	no	no	yes	no	no
Jirousek–Shenoy Equation [40]	$H\left(m\right)=\sum_{x\subseteq X}Pl\_P_{m}\left(x\right)log_{2}\frac{1}{Pl\_P_{m}\left(x\right)}+\sum_{A\subseteq X}m\left(A\right)log_{2}\left(\left|A\right|\right)$	yes	yes	no	yes	yes	yes	no
Yager Equation [48]	$H_{y}\left(m\right)=-\sum_{A\subseteq X}m\left(A\right)log_{2}Pl\left(A\right)$	no	no	no	no	yes	yes	no
Hohle Equation [49]	$H_{o}\left(m\right)=-\sum_{A\subseteq X}m\left(A\right)log_{2}Bel\left(A\right)$	no	no	no	no	yes	yes	no
Pal et al. Equation [50]	$H_{b}\left(m\right)=\sum_{A\subseteq X}m\left(A\right)log_{2}\left(\frac{\left|A\right|}{m(A)}\right)$	yes	yes	yes	yes	yes	yes	no

**Table 2:** Proposed measure Q(m) with *A*.

Cases	Q(m)
$A=\left\{1\right\}$	1.4285
$A=\left\{1,2\right\}$	1.5351
$A=\left\{1,2,3\right\}$	1.6821
$A=\left\{1,\ldots ,4\right\}$	1.8551
$A=\left\{1,\ldots ,5\right\}$	2.0476
$A=\left\{1,\ldots ,6\right\}$	2.2557
$A=\left\{1,\ldots ,7\right\}$	2.4765
$A=\left\{1,\ldots ,8\right\}$	2.7085
$A=\left\{1,\ldots ,9\right\}$	2.9500
$A=\left\{1,\ldots ,10\right\}$	3.2002
$A=\left\{1,\ldots ,11\right\}$	3.4580
$A=\left\{1,\ldots ,12\right\}$	3.7228
$A=\left\{1,\ldots ,13\right\}$	3.9941
$A=\left\{1,\ldots ,14\right\}$	4.2731

**Table 3:** Results of different uncertainty measures of Example 3.

BPAs	Deng	Pal et al.	Dubois–Prade	Proposed Measure
$m_{1}$	2.5559	1.9710	1	1.4710
$m_{2}$	2.5559	1.9710	1	1.6376

**Table 4:** Conflict data from sensors.

BPAs	$\left\{F_{1}\right\}$	$\left\{F_{2}\right\}$	$\left\{F_{3}\right\}$	$\left\{F_{1},F_{3}\right\}$
$S_{1}:m_{1}$	0.41	0.29	0.30	0.00
$S_{2}:m_{2}$	0.00	0.90	0.10	0.00
$S_{3}:m_{3}$	0.58	0.07	0.00	0.35
$S_{4}:m_{4}$	0.55	0.10	0.00	0.35
$S_{5}:m_{5}$	0.60	0.10	0.00	0.30

**Table 5:** Fusion results with different combination rules.

	$m_{1},m_{2}$	$m_{1},m_{2},m_{3}$	$m_{1},m_{2},m_{3},m_{4}$	$m_{1},m_{2},m_{3},m_{4},m_{5}$
Dempster’s rule [7]	$m(\left\{F_{1}\right\})=0$ $m(\left\{F_{2}\right\})=0.8969$ $m(\left\{F_{3}\right\})=0.1031$ $m(\left\{F_{1},F_{3}\right\})=0$	$m(\left\{F_{1}\right\})=0$ $m(\left\{F_{2}\right\})=0.6575$ $m(\left\{F_{3}\right\})=0.3425$ $m(\left\{F_{1},F_{3}\right\})=0$	$m(\left\{F_{1}\right\})=0$ $m(\left\{F_{2}\right\})=0.3321$ $m(\left\{F_{3}\right\})=0.6679$ $m(\left\{F_{1},F_{3}\right\})=0$	$m(\left\{F_{1}\right\})=0$ $m(\left\{F_{2}\right\})=0.1422$ $m(\left\{F_{3}\right\})=0.8578$ $m(\left\{F_{1},F_{3}\right\})=0$
Yager’s rule [55]	$m(\left\{F_{1}\right\})=0$ $m(\left\{F_{2}\right\})=0.2610$ $m(\left\{F_{3}\right\})=0.0300$ $m(\left\{F_{1},F_{3}\right\})=0$ $m(\left\{X\right\})=0.7090$	$m(\left\{F_{1}\right\})=0.4112$ $m(\left\{F_{2}\right\})=0.0679$ $m(\left\{F_{3}\right\})=0.0105$ $m(\left\{F_{1},F_{3}\right\})=0.2481$ $m(\left\{X\right\})=0.2622$	$m(\left\{F_{1}\right\})=0.6508$ $m(\left\{F_{2}\right\})=0.0330$ $m(\left\{F_{3}\right\})=0.0037$ $m(\left\{F_{1},F_{3}\right\})=0.1786$ $m(\left\{X\right\})=0.1339$	$m(\left\{F_{1}\right\})=0.7732$ $m(\left\{F_{2}\right\})=0.0167$ $m(\left\{F_{3}\right\})=0.0011$ $m(\left\{F_{1},F_{3}\right\})=0.0938$ $m(\left\{X\right\})=0.1152$
Mruphy’s rule [56]	$m(\left\{F_{1}\right\})=0.0964$ $m(\left\{F_{2}\right\})=0.8119$ $m(\left\{F_{3}\right\})=0.0917$ $m(\left\{F_{1},F_{3}\right\})=0$	$m(\left\{F_{1}\right\})=0.4619$ $m(\left\{F_{2}\right\})=0.4497$ $m(\left\{F_{3}\right\})=0.0794$ $m(\left\{F_{1},F_{3}\right\})=0.0090$	$m(\left\{F_{1}\right\})=0.8362$ $m(\left\{F_{2}\right\})=0.1147$ $m(\left\{F_{3}\right\})=0.0410$ $m(\left\{F_{1},F_{3}\right\})=0.0081$	$m(\left\{F_{1}\right\})=0.9620$ $m(\left\{F_{2}\right\})=0.0210$ $m(\left\{F_{3}\right\})=0.0138$ $m(\left\{F_{1},F_{3}\right\})=0.0032$
Deng et al. method [54]	$m(\left\{F_{1}\right\})=0.0964$ $m(\left\{F_{2}\right\})=0.8119$ $m(\left\{F_{3}\right\})=0.0917$ $m(\left\{F_{1},F_{3}\right\})=0$	$m(\left\{F_{1}\right\})=0.4974$ $m(\left\{F_{2}\right\})=0.4054$ $m(\left\{F_{3}\right\})=0.0888$ $m(\left\{F_{1},F_{3}\right\})=0.0084$	$m(\left\{F_{1}\right\})=0.9089$ $m(\left\{F_{2}\right\})=0.0444$ $m(\left\{F_{3}\right\})=0.0379$ $m(\left\{F_{1},F_{3}\right\})=0.0089$	$m(\left\{F_{1}\right\})=0.9820$ $m(\left\{F_{2}\right\})=0.0039$ $m(\left\{F_{3}\right\})=0.0107$ $m(\left\{F_{1},F_{3}\right\})=0.0034$
Zhang et al. method [57]	$m(\left\{F_{1}\right\})=0.0964$ $m(\left\{F_{2}\right\})=0.8119$ $m(\left\{F_{3}\right\})=0.0917$ $m(\left\{F_{1},F_{3}\right\})=0$	$m(\left\{F_{1}\right\})=0.5681$ $m(\left\{F_{2}\right\})=0.3319$ $m(\left\{F_{3}\right\})=0.0929$ $m(\left\{F_{1},F_{3}\right\})=0.0084$	$m(\left\{F_{1}\right\})=0.9142$ $m(\left\{F_{2}\right\})=0.0395$ $m(\left\{F_{3}\right\})=0.0399$ $m(\left\{F_{1},F_{3}\right\})=0.0083$	$m(\left\{F_{1}\right\})=0.9820$ $m(\left\{F_{2}\right\})=0.0034$ $m(\left\{F_{3}\right\})=0.0115$ $m(\left\{F_{1},F_{3}\right\})=0.0032$
The proposed method	$m(\left\{F_{1}\right\})=0.1648$ $m(\left\{F_{2}\right\})=0.7796$ $m(\left\{F_{3}\right\})=0.0556$ $m(\left\{F_{1},F_{3}\right\})=0$	$m(\left\{F_{1}\right\})=0.9417$ $m(\left\{F_{2}\right\})=0.0250$ $m(\left\{F_{3}\right\})=0.0322$ $m(\left\{F_{1},F_{3}\right\})=0.0011$	$m(\left\{F_{1}\right\})=0.9756$ $m(\left\{F_{2}\right\})=0.0039$ $m(\left\{F_{3}\right\})=0.0176$ $m(\left\{F_{1},F_{3}\right\})=0.0029$	$m(\left\{F_{1}\right\})=0.9849$ $m(\left\{F_{2}\right\})=0.0014$ $m(\left\{F_{3}\right\})=0.0105$ $m(\left\{F_{1},F_{3}\right\})=0.0032$

**Table 6:** Fusion results of Q(m) with different number of sensor reports.

$m_{1},m_{2}$	$m(\left\{F_{1}\right\})=0.1648$ $m(\left\{F_{2}\right\})=0.7796$ $m(\left\{F_{3}\right\})=0.0556$ $m(\left\{F_{1},F_{3}\right\})=0$	$F_{2}$
$m_{1},m_{2},m_{3}$	$m(\left\{F_{1}\right\})=0.9417$ $m(\left\{F_{2}\right\})=0.0250$ $m(\left\{F_{3}\right\})=0.0322$ $m(\left\{F_{1},F_{3}\right\})=0.0011$	$F_{1}$
$m_{1},m_{2},m_{3},m_{4}$	$m(\left\{F_{1}\right\})=0.9756$ $m(\left\{F_{2}\right\})=0.0039$ $m(\left\{F_{3}\right\})=0.0176$ $m(\left\{F_{1},F_{3}\right\})=0.0029$	$F_{1}$
$m_{1},m_{2},m_{3},m_{4},m_{5}$	$m(\left\{F_{1}\right\})=0.9849$ $m(\left\{F_{2}\right\})=0.0014$ $m(\left\{F_{3}\right\})=0.0105$ $m(\left\{F_{1},F_{3}\right\})=0.0032$	$F_{1}$
